# Chemical Biology of Autophagy-Related Proteins With Posttranslational Modifications: From Chemical Synthesis to Biological Applications

**DOI:** 10.3389/fchem.2020.00233

**Published:** 2020-04-03

**Authors:** Yu Luo, Chen Jiang, Lihua Yu, Aimin Yang

**Affiliations:** School of Life Sciences, Chongqing University, Chongqing, China

**Keywords:** autophagy, autophagy-related proteins, posttranslational modification, native chemical ligation, expressed protein ligation, Sortase A-mediated protein ligation, hydrazide-based native chemical ligation

## Abstract

Macroautophagy (hereafter referred to as autophagy) is an evolutionarily conserved lysosomal degradation pathway in all eukaryotic cells, which is critical for maintaining cell homeostasis. A series of autophagy-related (ATG) proteins are involved in the regulation of autophagy. The activities of ATG proteins are mainly modulated by posttranslational modifications (PTMs), such as phosphorylation, lipidation, acetylation, ubiquitination, and sumoylation. To tackle molecular mechanisms of autophagy, more and more researches are focusing on the roles of PTMs in regulation of the activity of ATG proteins and autophagy process. The protein ligation techniques have emerged as powerful tools for the chemical engineering of proteins with PTMs, and provided effective methods to elucidate the molecular mechanism and physiological significance of PTMs. Recently, several ATG proteins with PTM were prepared by protein ligation techniques such as native chemical ligation (NCL), expressed protein ligation (EPL), peptide hydrazide-based NCL, and Sortase A-mediated ligation (SML). More importantly, the synthesized ATG proteins are successfully used to probe the mechanism of autophagy. In this review, we summarize protein ligation techniques for the preparation of ATG proteins with PTMs. In addition, we highlight the biological applications of synthetic ATG proteins to probe the autophagy mechanism.

## Introduction

**Figure 1:**
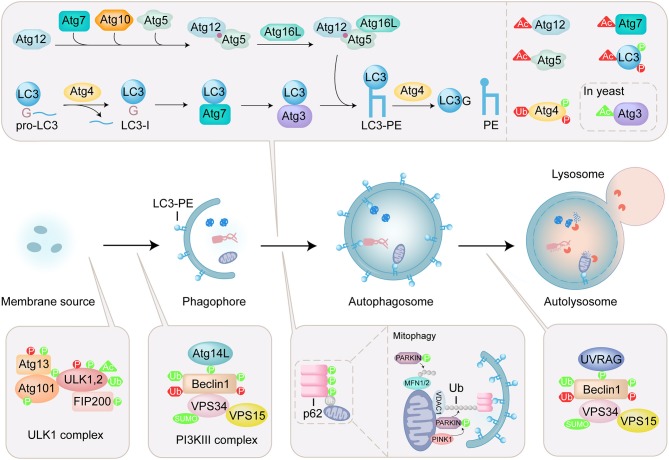
Overview of autophagy process and regulation of ATG proteins by PTMs. Autophagy is a lysosomal degradation pathway that sequesters and engulfs cytoplasmic targets. The induction of autophagy is initiated by ULK complex which contains ULK1/2, autophagy-related (ATG) 13, focal adhesion kinase family interacting protein of 200 kDa (FIP200), and ATG101. The PI3KIII complex containing Beclin1, ATG14L, vacuolar protein sorting (VPS) 34, and vacuolar protein sorting 15 (VPS15) is activated by ULK1 initiates formation of phagophore. Two ubiquitin-like (Ubl) conjugation systems (Atg12 conjugation system and LC3 lipidation system) mediate the elongation and closure of the autophagosome. The maturation of the autophagosome and the fusion with the lysosome are mediated by the PI3KIII complex containing Beclin1, UV radiation resistance-associated gene product (UVRAG), vacuolar protein sorting (VPS) 34, and vacuolar protein sorting 15 (VPS15). Autophagy receptor, such as p62, specifically recognizes ubiquitinated substrates and binds to LC3-PE. In mitophagy, PINK1 is stabilized on the outer mitochondrial membrane and activated by autophosphorylation, triggering Parkin recruitment and activation. Then mitochondrial surface proteins, such as voltage-dependent anion-selective channel protein 1 (VDAC1) and mitofusins (MFN1/2) are ubiquitinated by Parkin, modulating mitochondrial dynamics. The PTMs including phosphorylation (P), acetylation (Ac), ubiquitination (Ub) and sumoylation (SUMO) are shown on ATG proteins. PTMs that positively regulate the autophagy process are in green, and those that negatively regulate the autophagy process are in red.

Macroautophagy, hereafter referred to as autophagy, is an evolutionarily conserved lysosomal degradation pathway that removes cytoplasmic components including protein aggregates, damaged organelles and intracellular pathogens in all eukaryotic cells (Feng et al., 2014; Ohsumi, 2014). The hallmark of autophagy is the appearance of cytoplasmic double-membrane vesicles, termed autophagosome that sequesters and engulfs cytoplasmic targets. After fusion with lysosomes, the cargo of autophagosome is exposed to lysosomal hydrolases for degradation (Figure 1). Autophagy plays an important role in maintaining cell homeostasis and regulating cellular energy metabolism. The dysregulation of autophagy is closely related to disease, including neurodegenerative diseases, cancer, and aging (Menzies et al., 2015; Santana-Codina et al., 2017; Hansen et al., 2018; Levine and Kroemer, 2019). Because of its great significance in physiological function, autophagy won the 2016 Nobel Prize in Physiology or Medicine (Levine and Klionsky, 2017).

A series of autophagy-related (ATG) proteins are involved in the regulation of autophagy (Mizushima et al., 2011). Specially, microtubule-associated protein light chain 3 (LC3), a mammalian homolog of yeast Atg8, is involved in both biogenesis of autophagosomes and recruitment of autophagic cargos. LC3 undergoes carboxyl-terminal cleavage and subsequent PE conjugation (Figure 1). Briefly, newly synthesized proLC3 is processed by an endogenous cysteine protease Atg4 to expose a C-terminal glycine, named LC3(1-120) or LC3-I. In the presence of Atg7, Atg3, and Atg5-Atg12:Atg16 (E1-like, E1-like, and E3-like enzyme, respectively), LC3-I is conjugated to phosphoethanolamine (PE) by the ubiquitin-like conjugation reaction to produce lipidated form, LC3-PE or LC3-II (Ichimura et al., 2000; Geng and Klionsky, 2008). Atg4 is also involved in the recycling of LC3 by deconjugation of LC3-PE (Satoo et al., 2009; Yu et al., 2012). In selective autophagy, autophagy receptors are responsible for the binding with autophagic cargos. p62 is an important mitophagy receptor involved in the removal of ubiquitinated mitochondria (Figure 1) (Ichimura et al., 2008; Peng et al., 2017). p62 specifically recognizes ubiquitinated substrates through its ubiquitin-associated (UBA) domain, and binds to LC3 via LC3-interacting region (LIR), thereby selectively engulfs the substrates into autophagosome.

The activities of ATG proteins are mainly modulated by posttranslational modifications (PTMs), such as phosphorylation, lipidation, acetylation, ubiquitination, and sumoylation (Figure 1) (Wani et al., 2015; Xie et al., 2015). These modifications play an important role in controlling the fate of the ATG proteins and the process of autophagy. Thus, uncovering the roles of PTMs in regulating the function of ATG proteins is crucial for understanding the mechanisms of autophagy. Over the past few years, protein ligation techniques have emerged as powerful approaches for the chemical engineering of proteins with PTMs, and provided effective methods to elucidate the molecular mechanism and physiological significance of PTMs (Hackenberger and Schwarzer, 2008; Bondalapati et al., 2016; Conibear et al., 2018). Recently, several ATG proteins with PTMs were obtained by protein ligation techniques and used to tackle the autophagy issues. In this review, protein ligation techniques for the preparation of ATG proteins are summarized and biological application of synthetic ATG proteins for the elucidation of autophagy mechanism are highlighted.

## Protein Ligation

**Figure 2:**
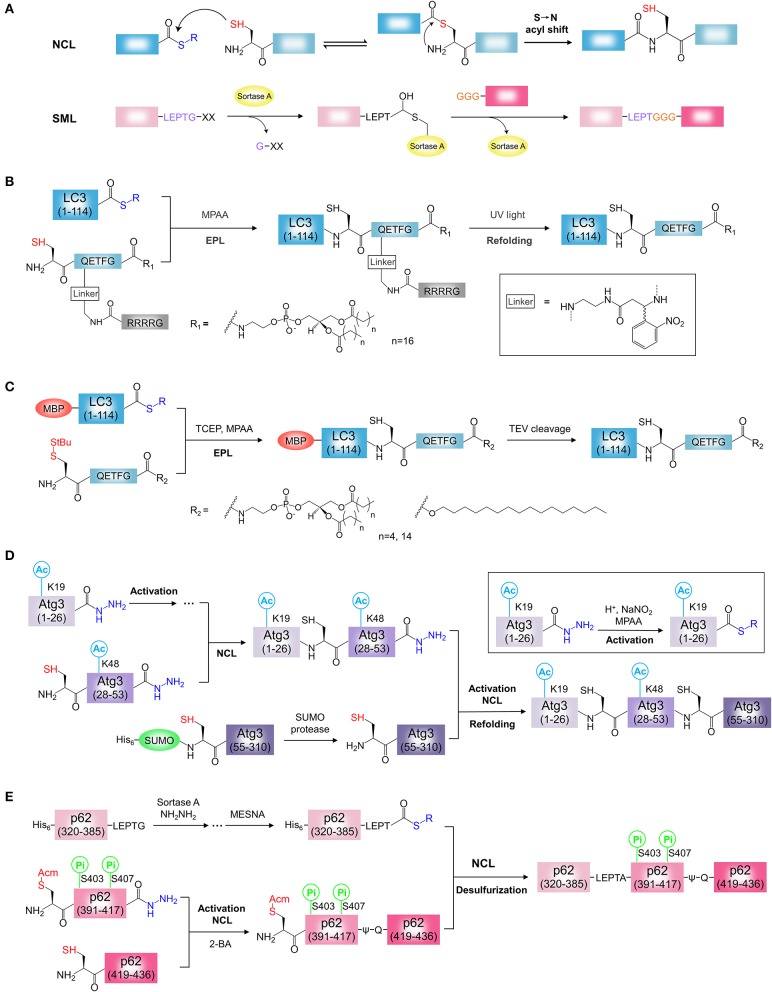
Chemical synthesis of ATG proteins with PTM. **(A)** The mechanisms of NCL and SML. NCL, native chemical ligation; SML, Sortase A-mediated ligation. **(B)** Synthesis of LC3-PE using light activatable solubilizing side chain strategy. **(C)** Synthesis of LC3-lipid conjugates using MBP tag assisted solubilization strategy. **(D)** Synthesis of acetylated Atg3 by sequential hydrazide-based NCL. **(E)** Synthesis of phosphorylated p62 by SML and hydrazide-based NCL. 2-Bromoacetamide, 2-BA.

In the 1990s, Kent and co-workers proposed the native chemical ligation (NCL) for the first time and used activated thioesters for protein synthesis (Dawson et al., 1994). In NCL, the sulfhydryl functionality of an N-terminal Cys residue of an unprotected peptide selectively attacks the C-terminal thioester of another unprotected peptide to form a thioester intermediate, which is a reversible process. Then the intermediate undergoes the rearrangement by spontaneously intra-molecular S → N acyl shift, which is an irreversible process, and a native peptide bond is formed between two peptides (Figure 2A) (Dawson and Kent, 2000). The NCL linking unprotected polypeptides has been emerging to be one of most effective methods in the field of protein engineering (Cistrone et al., 2019).

In NCL, a Cys residue is required at the ligation site to achieve chemoselective reaction. However, Cys is an uncommon amino acid in proteins. The combination of NCL with desulfurization techniques gives access to challenging proteins without Cys (Jin and Li, 2018). In the early studies, Pd/Al_2_O_3_ and Raney nickel were used to convert Cys into Ala after NCL, which expands the Cys-based peptide ligation repertoire to “Ala ligation” (Yan and Dawson, 2001; Pentelute and Kent, 2007). In these metal-based desulfurization methods, however, excessive use of nickel causes epimerization of secondary alcohols and the reduction of thiols, thioethers, and thioesters. To overcome the disadvantages of metal-based desulfurization methods, Danishefsky and co-workers developed a mild and highly versatile metal-free radical-based desulfurization with TCEP (tris(2-carboxyethyl)phosphine)/VA-044/t-BuSH (Wan and Danishefsky, 2007). Recently, Li and co-workers developed a P-B desulfurization approach which involves the use of common reagents, TCEP/NaBH_4_, or TCEP/LiBEt_3_H (Jin et al., 2017).

In addition, other thiol-derived amino acids have been used as Cys surrogates in NCL, and then converted into the native amino acid residues following desulfurization step (Kulkarni et al., 2018). Another Cys surrogate selenocysteine (Sec) has been used to perform NCL, and postligation deselenization allows preparation of proteins containing natural amino acids (Metanis et al., 2010; Mitchell et al., 2017). Overall, NCL-based extension methods have been applied to the synthesis of a growing number of proteins (Malins and Payne, 2014; Yan et al., 2018; Agouridas et al., 2019).

The chemical synthesis of protein is largely restricted by the length of peptides accessible by solid-phase peptide synthesis (SPPS). To expand the application of NCL, Muri, and co-workers proposed the expressed protein ligation (EPL) based on a protein splicing technology (Muir, 2003; Shah and Muir, 2014), and the field of protein chemical synthesis was pushed to a climax. In EPL, target protein is fused to the N-terminus of intein and prepared by the recombinant protein technology. The first step in the splicing process involves an N → S acyl shift in which the target protein is transferred to the sulfhydryl functionality of Cys. Then, a sulfhydryl reagent (such as 2-mercapoethanesulfonate, MESNA) is added for cleavage of the engineered intein to obtain the protein α-thioester which is ready to undergo NCL with a synthetic peptide containing N-terminal Cys. In short, the EPL has greatly expanded the application of protein chemical ligation to solve more complex biological problems (Vila-Perello and Muir, 2010; Holt and Muir, 2015).

The synthesis of peptide thioesters remains challenging using Fmoc chemistry. To overcome the limitation, Liu's group proposed the peptide hydrazide-based NCL for protein chemical synthesis (Fang et al., 2011; Zheng et al., 2013). In this method, the thioester equivalent, peptide hydrazide is cleanly oxidized to peptide azide by sodium nitrite (NaNO_2_), and the addition of sulfhydryl further promotes the thioesterification of peptide azide. In addition, the sequential ligation in the N-to-C direction can be achieved by conducting the oxidation step for the first peptide hydrazide before adding the second peptide that possesses both an N-terminal Cys and a C-terminal hydrazide. Unlike peptide thioesters, peptide hydrazide can be easily prepared by Fmoc-based SPPS. The peptide hydrazide-based NCL has been successfully applied to the chemical synthesis of several proteins (Li et al., 2014; Pan et al., 2014; Lan et al., 2016; Shi et al., 2017). Recently, Flood and co-workers reported a new method for activation of peptide hydrazide under the mild condition (Flood et al., 2018). In this method, C-terminal peptide hydrazides are activated in solution with stoichiometric acetyl acetone and converted into their corresponding thioesters via an acyl pyrazole intermediate. This method is compatible with N-terminal thiazolidines (Thz) and provides a general approach for the production of C-terminal peptide thioesters.

Alternatively, enzyme-mediated ligations such as Sortase A-mediated ligation (SML) and thiol-independent peptide ligations techniques such as serine/threonine ligation (STL) and α-ketoacid-hydroxylamine (KAHA) ligation were developed and applied for protein chemical synthesis (Bode, 2017; Schmidt et al., 2017; Liu and Li, 2018; Thompson and Muir, 2020). Sortase A is a calcium-dependent transpeptidase isolated from Staphylococcus aureus in the 1990s (Mazmanian et al., 1999). Sortase A specifically binds to the LPXTG sequence (X is any amino acid) at the C-terminus of the protein, and cleaves the peptide bond between threonine and glycine, leading to the formation of a thioester intermediate at the Cys184 in Sortase A. The resulting thioester intermediate is attacked by N-terminal glycine (1-5 Gly residues) unprotected peptide, and then a natural peptide bond is formed between the nucleophilic group and threonine, and Gly in the LPXTG sequence is cleaved (Figure 2A) (Mao et al., 2004; Ling et al., 2012; Staus et al., 2018). SML is independent of Cys and successfully applied to the production of proteins (Policarpo et al., 2014; Wang et al., 2017; Pishesha et al., 2018; Staus et al., 2018).

As the demand for synthesis of larger protein or protein carrying multiple PTMs, the proteins have to be divided into multiple segments. In multi-segment ligations, several ligations need to be performed to afford the target. To this end, the middle segment must contain N-terminal Cys or Cys surrogate and C-terminal thioester or thioester surrogate, and undergoes multiple ligations. Multiple segments can be obtained by SPPS or protein engineering and assembled in both C to N and N to C terminal directions. To avoid cyclization of middle segments during ligation, N-terminal Cys of middle segment is protected or the thioester is inactive form, which depends on assembly directions of target protein. For C to N direction sequential chemical ligation, the first ligation is carried out between C-terminal thioester of middle segment and N-terminal Cys of C-terminal segment. For this purpose, the N-terminal Cys of middle segment must be protected before ligation. After first ligation, the protected group is removed to exposure free Cys for second ligation. Thz (1,3-thiazolidine-4-carboxo) protecting group of N-terminal Cys or the S-protecting group Acm is widely used to mask the N-terminal Cys of middle segment. Because of their removal under mild condition, Thz and Acm group are applied to the sequential ligation of multi-segments for the preparation of lots of proteins (Bang and Kent, 2004; Piontek et al., 2009; Fauvet et al., 2013; Maity et al., 2016). For N to C direction sequential chemical ligation, similarly, the C-terminal thioester of middle segment must be protected before ligation. The thioester precursor has no reactivity, and its thioester reactivity can be recovered after activation. So, thioester precursor, such as hydrazide, is a good choice for the C to N sequential ligation. Middle segment hydrazide has been successfully used to produce a number of large proteins (Zheng et al., 2013; Tang et al., 2015, 2017). Significantly, in multi-segment protein ligation, each intermediate must be separated and purified for the next reaction. One-pot ligation skips the isolation and purification of intermediate products, and improves the yield and reduces the reaction time. The recent review provided the progress on one-pot multi-segment condensation strategies (Zuo et al., 2019).

## Chemical Synthesis of ATG Proteins

LC3-PE is the first semi-synthetic ATG protein by protein ligation. Chemical synthesis of lipidated proteins still remains a challenging task due to poor solubility of lipidated proteins (Yang et al., 2015; Mejuch and Waldmann, 2016; Takahara and Kamiya, 2020). To overcome this problem, two independent groups reported different synthesis strategies for the preparation of LC3-PE by EPL (Huang et al., 2013; Yang et al., 2013). Considering the expression efficiency and reactivity of protein thioester, the Ala114-Ser115 bond was selected as the ligation site in two reports. By retrosynthetic analysis, the protein thioester, and lipidated peptide need to be obtained for ligation (Figures 2B,C). For the preparation of lipidated peptide, Huang et al. developed a novel removable solubilizing side chain to facilitate the solubility of the lipidated peptide (Huang et al., 2013). Briefly, the fully protected hexapeptide was assembled by standard Fmoc SPPS employing a special building block, ortho-nitrobenzyl modified Gln. At the end of the peptide, the Alloc group on the side chain of Gln was removed in the presence of catalytic amount of palladium (0) and then the liberated amino group was assembled to the poly Arg (Arg4) tag capped with a Boc-Gly-OH residue on the N terminus. After release from the resin, the protected peptide was condensed with 1,2-distearoyl-sn-glycero-3-phosphoethanolamine (DSPE) and then the deprotection step afforded the final lipidated peptide suitable for the EPL. The ligation of PE-modified peptide with LC3 (1-114) thioester from intein strategy was performed successfully under detergent-free conditions. The Arg4 tag and the photosensitive linker were removed by UV irradiation. After refolding, functional LC3-DSPE was obtained (Figure 2B).

Yang et al. developed MBP tag assisted solubilisation strategy for the preparation of LC3-PE protein (Figure 2C) (Yang et al., 2013). The MBP tag was used to facilitate solubilization of the lipidated protein, which enables the ligation under folding conditions. The peptide with PE modification was synthesized using the chlorotrityl resin by standard Fmoc SPPS. After release from the resin, the peptide was coupled with 1,2-dipalmitoyl-sn-glycero-3-phosphoethanolamine (DPPE) in the presence of pentafluorphenyl trifluoracetate (PFP-TFA). Removing all acid sensitive protecting groups by a high concentration of TFA afforded the desired the DPPE modified peptide. Finally, the ligation of MBP-LC3 (1-114)-thioester and the lipidated peptide was performed under folding conditions in presence of detergent β-octylglucoside and MPAA. The resulting MBP-LC3-PE was soluble and stable in buffer without detergents. Alternatively, LC3-PE was obtained by removal of the MBP tag by TEV protease cleavage. Using this strategy, a series of LC3-lipid conjugates, such as LC3-DHPE (1,2-dihexanoyl-sn-glycero-3-phosphoethanolamine) and LC3-C16 (1-hexadecanol), were obtained (Figure 2C) (Yang et al., 2017a,b). This strategy seems to be simpler and more efficient, the synthesis procedure of PE modified peptide is not complicated and the refolding step of lipidated proteins is unnecessary. In this strategy, however, the detergent was used for ligation and need to be removed after ligation. Overall, both strategies are effective for the synthesis of homogeneous LC3-PE. In addition, light activatable solubilizing side chain strategy and MBP tag assisted solubilisation strategy would provide general approaches for the synthesis of hydrophobic proteins, such as lipidated proteins or membrane proteins (Li J. B. et al., 2017).

Recently, Li and co-workers synthesized the K19/K48-diacetylated Atg3 protein by sequential hydrazide-based NCL (Figure 2D) (Li Y. T. et al., 2017). Considering the protein structure and the possibility of peptide production, the Gly26-Gln27 and Ser53-Ser54 were chosen as the ligation sites with Gln27 to Cys27 mutation, Ser54 to Cys54 mutation for two ligations. By retrosynthetic analysis, Atg3(1-310) was divided into three protein segments, Atg3(1-26), Atg3(27-53), and Atg3(54-310) (Figure 2D). The building block, Fmoc-N'-acetyl-L-lysine, was used to generate the acetylated Atg3(1-26) and Atg3(27-53) peptide hydrazides by SPPS. The third segment, Atg3(54-310) was prepared by recombinant protein expression using SUMO tag fusion approach, and the SUMO tag was removed by the SUMO-specific protease with excellent efficiency. To obtain the full length of diacetylated Atg3 protein, N to C direction sequential chemical ligation was performed by hydrazide-based NCL. Firstly, the segments, Atg3(1-26) and Atg3(27-53) peptide hydrazides were ligated by NCL after the activation of Atg3(1-26) peptide hydrazide. The resulting peptide, in the following step, was ligated with the third segment Atg3(54-310) in the same way to obtain the full-length peptide. Further folding and purification afforded the semi-synthetic functional Atg3 K19ac-K48ac (Figure 2D).

The phosphorylated p62 protein was obtained by SML and hydrazide-based NCL (Figure 2E) (Tan et al., 2017). To minimize the complexity of synthesis procedures, a truncated p62(320-436) with UBA domain and LIR was chosen as the synthetic target. By retrosynthetic analysis, p62(320-436) was divided into three protein segments, p62(320-385) tagged by Sortase A cleavage sequence LPETG, p62(390-417), and p62(418-436) (Figure 2E). The phosphorylation sites are localized at the segment p62(390-417). The segment p62(320-385)-LPETG was obtained by recombinant expression and then converted to the hydrazide form by Sortase A-mediated hydrazinolysis method. The building block, Fmoc-Ser(HPO_3_Bzl)-OH, was used to generate the phosphorylated p62(390-417) hydrazide with S403Pi and S407Pi. The segment p62(418-436) was synthesized by SPPS. To obtain phosphorylated p62, three segments were assembled in C to N direction using sequential chemical ligation strategy. The phosphorylated segment p62(390-417) hydrazide was activated and ligated with p62(418-436). To avoid the byproduct, the Cys at ligation site was capped with 2-bromoacetamide to form ψ-Gln418. By removal of S-protecting group Acm, thiol group of N-terminal Cys in was exposed. Then, the resulting peptide and the segment p62(320-385)-LPETG was ligated by EPL after the activation of p62(320-385)-LPETG peptide hydrazide. The Cys390 incorporated by second ligation was convert to Ala by final desulfurization. After folding, the desired phosphorylated p62 proteins were obtained (Figure 2E).

## Probing the Autophagy Using Synthetic ATG Proteins

**Figure 3:**
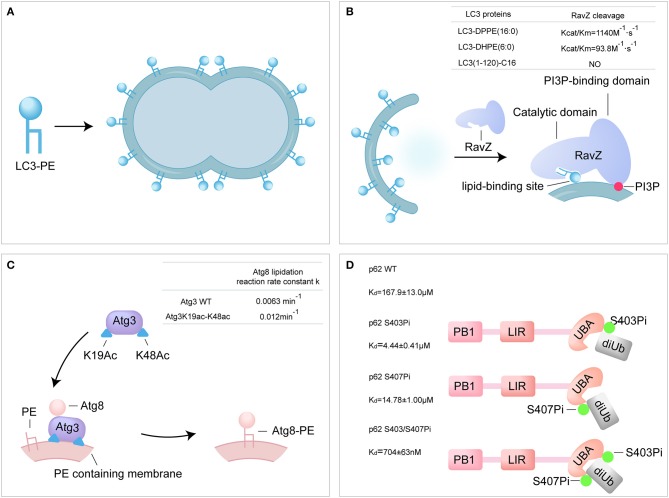
Probing the autophagy using synthetic ATG proteins. **(A)** Model of LC3-PE promoting liposome tethering and fusion. **(B)** Model of RavZ-mediated LC3-PE deconjugation on autophagosome membrane. RavZ targets to autophagosome membrane by interaction of its C-terminal domain with PI3P and binds PE through its lipid-binding site. **(C)** Model of acetylated Atg3 facilitating Atg8 lipidation in yeast. Acetyl groups of Atg3 promote Atg3 membrane binding and recruit Atg8 to facilitate Atg8 lipidation. **(D)** The bidentate binding model of phosphorylated p62 binding with K63 diUb. Phosphorylated S403 and S407 sites of p62 bind to different epitopes on the ubiquitin chain.

Previous attempts to produce LC3-PE have relied on maleimide-coupling strategy or *in vitro* conjugation system. In maleimide-coupling strategy, C-terminal Gly of LC3-I is mutated to Cys and conjugated to the maleimide PE by thiol-maleimide “click” reactions. The resulting LC3-PE, however, is not native protein and could not mimic the behavior of native LC3-PE (Weidberg et al., 2011). *In vitro* conjugation of LC3-I with PE was performed using E1-like and E2-like enzymes, Atg7 and Atg3 in presence of PE containing liposomes. Unfortunately, i*n vitro* conjugation approach affords LC3-PE in a protein-lipid mixture (Nakatogawa et al., 2007). *In vitro* cleavage assay showed that the semisynthetic LC3-PE was processed by Atg4B to generate LC3-I. The cleavage of semisynthetic LC3-PE protein was significantly impaired by the Atg4B inhibitor, methyl 7-deshydroxypyrogallin-4-carboxylate (MDC) (Yang et al., 2013). Furthermore, the liposome assay showed that the semisynthetic LC3-PE mediates membrane tethering and fusion (Figure 3A). The semi-synthesis of LC3-PE provides a new research method for the autophagy.

RavZ, a *Legionella pneumophila* effector, functions as a cysteine protease and irreversibly delipidates LC3-PE to inhibit autophagosome formation (Choy et al., 2012). RavZ targets the autophagosome via the PI3P-binding domain (Horenkamp et al., 2015). Unlike endogenous ATG4B, RavZ only cleaves the PE-modified LC3, rather than proLC3. Moreover, RavZ mediated the deconjugation of LC3-PE to generate LC3(1-119) that is not able to be reused, while ATG4B cleaved LC3–PE to produce LC3-I. However, how RavZ recognizes and deconjugates LC3–PE remains elusive due to lack of various modified LC3 proteins. The various semisynthetic LC3 proteins were used to define the structure-function relationship of RavZ. The cleavage assay showed that RavZ displays a 12-fold reduction of catalytic efficiency toward LC3–DHPE compared to LC3-DPPE and no activity for LC3-C16, which suggests that RavZ preferred LC3-PE with long fatty acid chains. Further experiments have demonstrated the working model of RavZ, in which RavZ contains a lipid-binding site for the recongnization of lipid part of LC3-PE (Figure 3B) (Pantoom et al., 2017; Yang et al., 2017b).

Atg3 is an E2-like enzyme and mediates the lipidation of Atg8 (Yamada et al., 2007; Nath et al., 2014). Acetylation of K19 and K48 of Atg3 was meditated by the acetyltransferase Esa1 and regulates the activity of autophagy (Yi et al., 2012). The semisynthetic Atg3K19ac-K48ac was used to address the mechanism of Atg3 acetylation regulating Atg8-PE lipidation (Li Y. T. et al., 2017). Firstly, *in vitro* Atg8 lipidation assay showed that the acetylated Atg3 facilitated the lipidation of Atg8 in the presence of Atg7 and PE-containing liposome (lipidation reaction rate constant: Atg3 WT 0.0063 min^−1^ vs. Atg3 K19ac-K48ac 0.012 min^−1^). The synthetic Atg3 K19ac-K48ac was able to pull down larger amount of Atg8 from yeast cell lysate compared to Atg3 wild type (WT). Furthermore, surface plasmon resonance (SPR) and microscale thermophoresis (MST) were used to quantify the binding affinity between Atg3 and Atg8, and the results showed that acetylation did not alter the interaction between Atg3 and Atg8 *in vitro*. Effect of acetylation on the interaction of Atg3 and liposome was further examined by liposome assay and the results showed that the acetylation could promote the interaction between Atg3 and PE-containing liposome. The possible explanation is that, the acetyl groups of Atg3 promote the binding of Atg3 to the PE-containing liposome membrane through hydrophobic or electrostatic interactions, and then regulate the Atg8 lipidation process (Li Y. T. et al., 2017). Overall, the synthetic acetylated Atg3 provides a new model for elucidating the role of acetylation in autophagy through enhancing the binding of Atg3 to membrane source for Atg8 lipidation (Figure 3C).

It is reported that the Ser403 and Ser407 of p62 are phosphorylated in UBA domain by autophagy-related Unc-51-like kinase 1 (ULK1) (Matsumoto et al., 2011; Lim et al., 2015). The phosphorylated p62 proteins were used to address the regulation of p62 phosphorylation. Firstly, binding affinities between various form of p62 and K63 di-ubiquitin were measured using SPR. The binding affinities of di-ubiquitin (K63-linked) to p62 S403Pi, p62 S407Pi, and p62 S403/407Pi were 4.4 ± 0.4 μM, 14.8 ± 1.0 μM, and 704 ± 63 nM, respectively, representing a 34, 11, or 240-fold enhancement over p62 WT (167.9 ± 13.0 μM). The bisphosphorylated p62 S403/407Pi showed an extreme strong affinity to di-ubiquitin compared to monophosphorylated form. In addition, the binding affinity of mimetic phosphorylated mutant p62 S403E-S407E and di-ubiquitin (K63-linked) was 46.6 ± 3.5 μM, much lower than natural biphosphorylated p62. This great difference in binding affinity suggests that p62 S403E-S407E is an inappropriate molecular tool for the analysis of the p62 phosphorylation. In conclusion, the molecular recognition of bisphosphorylated p62 and ubiquitin was proposed as a bidentate binding mechanism in selective autophagy (Figure 3D) (Tan et al., 2017).

## Synthesis of Phosphorylated Di-Ubiquitin and their Application in the Study of Mitophagy

Mitophagy is the selective degradation of damaged mitochondria by autophagic machinery and plays an important role in maintaining mitochondrial quality control and cellular homeostasis (Palikaras et al., 2018). In mitophagy, PINK1/Parkin pathway has been identified as a key mechanism to sense and label damaged mitochondria (Figure 1) (Jin and Youle, 2012). PTEN-induced kinase 1 (PINK1) accumulates on the outer membrane of damaged mitochondria and phosphorylates ubiquitin chains or Parkin Ub-like (UBL) domain to activate Parkin (Nguyen et al., 2016; Pickles et al., 2018). However, how phosphorylated ubiquitin chain activates Parkin is unclear (Kumar et al., 2017; Condos et al., 2018; Gladkova et al., 2018; Sauve et al., 2018).

**Figure 4:**
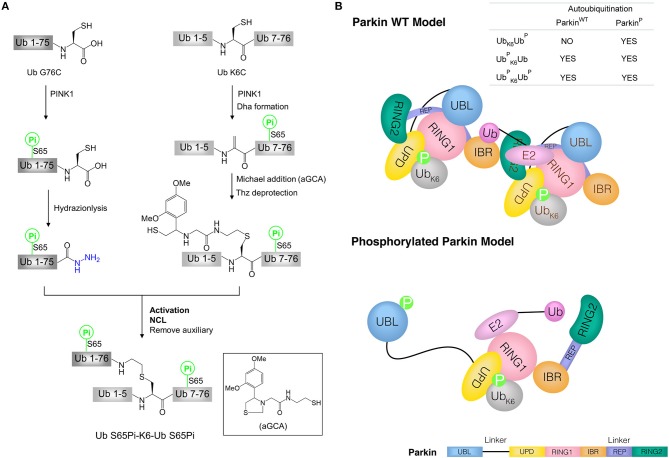
Synthesis of phosphorylated di-ubiquitin and their application in the study of mitophagy. **(A)** Synthesis of phosphorylated di-ubiquitin chain (K6-linked). **(B)** Models of Parkin WT and phosphorylated Parkin activated by di-ubiquitin. Parkin consists of an N-terminal UBL domain connected through linkers to four Zn^2+^-binding domains: unique parkin domain (UPD, also known as RING0), RING1 (containing an E2-binding site), in-between RING (IBR), and RING2 (catalytic domain). Parkin C-terminal RING-IBR-RING domains for E3 activity is masked by the Ubl domain. In Parkin wild type (WT) model, two molecules of Parkin cooperate to activate Parkin WT without the release of the UBL domain in the presence of Ub S65Pi-K6-Ub or Ub S65Pi-K6-Ub S65Pi. In phosphorylated Parkin model, phosphorylated UBL is released and catalytic RING2 domain achieves the activation of phosphorylated Parkin by Ub-K6-Ub S65Pi.

Liu and co-workers synthesized the phosphorylated K6-linked di-ubiquitin by hydrazide-based NCL (Figure 4A) (Pan et al., 2019). To apply this strategy, di-ubiquitin was divided into two segments, ubiquitin G76C (Ub G76C) and ubiquitin K6C (Ub K6C). Both segments were obtained by protein expression and the phosphorylated forms at Ser65 residue were produced by using recombinant kinase PINK1. Ub S65Pi hydrazide was prepared from Ub G76C by the Cys-promoted C-terminal hydrazinolysis method. Ub K6C S65Pi was converted to a Dha-modified intermediate through a bis-alkylation-elimination promoted by α, α′-di-bromo-adipyl (bis)amide. Then, Ub bearing an isopeptide bond mimic was obtained by Michael addition reaction of Dha group with glycyl cysteamine auxiliary (aGCA). In the next one-pot reaction, the Thz protecting group was removed to exposure the thiol group and amide group. Finally, Ub S65Pi hydrazide and Ub K6C (aGCA) S65Pi were ligated by hydrazide-based NCL to afford the biphosphorylated Ub S65Pi-K6-Ub S65Pi (Ub${}_{\text{K}6}^{\text{P}}$Ub^P^) after removal auxiliary group (Figure 4A). Using same strategy, mono phosphorylated derivatives, Ub-K6-Ub S65Pi (Ub_K6_Ub^P^), and Ub S65Pi-K6-Ub (Ub${}_{\text{K}6}^{\text{P}}$Ub), were synthesized successfully. The X-ray structure of Ub${}_{\text{K}6}^{\text{P}}$Ub^P^ showed the synthetic di-ubiquitin had the appropriate folded structures.

The synthetic di-ubiquitin (K6-linked) proteins were used to further investigate the mechanism by which phosphorylated ubiquitin chains activate Parkin and drive the elimination of impaired mitochondria. Since full-length human Parkin WT is used not only as an E3 enzyme, but also as a substrate to be autoubiquitinated, the ability of synthetic di-ubiquitin (K6-linked) to activate Parkin WT or phosphorylated Parkin was evaluated using Parkin autoubiquitination assay. Interestingly, in the presence of Ub, Uba1 (E1) and Ubch7 (E2), Ub S65Pi-K6-Ub and Ub S65Pi-K6-Ub S65Pi activated both of Parkin WT and phosphorylated Parkin, while Ub-K6-Ub S65Pi activated phosphorylated Parkin but not Parkin WT. Furthermore, E2 discharge experiment showed that Ub-K6-Ub S65Pi could not transfer the Ub thioester from E2~Ub to Parkin WT. Combination with previous works (Shiba-Fukushima et al., 2012; Koyano et al., 2014), the models of the activation of Parkin WT and phosphorylated Parkin by di-ubiquitin chains were proposed in mitophagy (Figure 4B) (Pan et al., 2019).

## Conclusions and Perspectives

Protein ligation techniques have emerged as powerful tools for the preparation of homogeneous ATG proteins with PTM. These synthetic proteins can be used to investigate protein-protein interactions and provide the insights into the roles of PTMs in the regulation of autophagy. Protein chemical synthesis has allowed the study of autophagy previously not possible through traditional cell biological and molecular biological tools.

Autophagy plays an important role in maintaining cell homeostasis and regulating cellular energy metabolism. In most cases, the activity of per ATG protein is modulated by multiple different PTMs (Figure 1) (Wani et al., 2015; Xie et al., 2015). Because of their efficiency and practicality, chemical ligation techniques would attract more and more attentions to elucidate the roles of PTM in regulation of autophagy. It is expected that the regulation of individual PTM or multiple PTMs per protein on autophagy will be addressed using synthetic ATG proteins.

## Author Contributions

YL and AY wrote the first draft of the manuscript. All authors contributed to manuscript revision, approved the final version and contributed to the conception of this work.

### Conflict of Interest

The authors declare that the research was conducted in the absence of any commercial or financial relationships that could be construed as a potential conflict of interest.
